# Supplementary material for: Selenium Compounds Affect Differently the Cytoplasmic Thiol/Disulfide State in Dermic Fibroblasts and Improve Cell Migration by Interacting with the Extracellular Matrix
Source: Antioxidants (Basel). 2024 Jan 26;13(2):159. doi: 10.3390/antiox13020159 (PMC10886037; doi:10.3390/antiox13020159)
Supplement: Supplementary file 1 [file antioxidants-13-00159-s001.zip › antioxidants-2798852-supplementary.pdf]

**Supplemental Figure S1.** A detailed view of the HyPer signal increase during the exposure to hydrogen peroxide for fibroblasts cultured in normal (empty circles) and high glucose (filled circles) conditions. Data correspond to the average ± SE of the same number of cells described in A1 and B1.

**Supplemental Figure S2. $LC_{50}$ of cell survival assays in CCD1068Sk cells in normal and high glucose with selenium compounds.** CCD1068Sk p10-25 cells were maintained in 5 (NG) or 25 (HG) mM glucose and treated with **A.** Sodium selenite (SS, $Na_2SeO_3$), (**B**) Selenocysteine (SeCys) and (**C**) Selenomethionine (SeMet), for 10 days. The percentage of live cells was quantified using vital trypan blue staining under brightfield microscopy with the Neubauer chamber, evaluating three independent assays in triplicate. Empty circles represent cells maintained in NG and filled circles in HG condition; these represent average ± SE. They were fitted to the equation $y = 100/((1 + X)/IC50)$ to estimate the $LC_{50}$. Data fit is shown by the solid line.

**Supplemental Figure S3. Effects of thioredoxin inhibitor on the recovery of HyPer biosensor in human fibroblasts treated with selenium compounds maintained in normal and high glucose.** Spontaneous HyPer signal recovery was measured right after the $H_2O_2$ removal, assigning 100% to this maximal signal at zero time in 5 (NG) and 25 (HG) mM glucose conditions, respectively. Data correspond to the observations in Figure 2 comparing the biosensor recovery in the presence of thioredoxin-1 inhibitor, PX-12. All the plots on the left correspond to NG-fibroblasts (A, C and E); All the plots on the right (**B**, **D** and **F**) correspond to HG-fibroblasts. In **A** and **B**, the effect of sodium selenite (SS); In **C** and **D**, selenocysteine (SeCys); and **F** and **F**, fibroblast treated with Selenomethionine (SeMet).

**Supplemental Figure S4. Effects of selenium compounds on the relative expression of selenoenzymes, redox enzymes and myofibroblast markers.** CCD1068Sk p10-17 cells were maintained in 5 (NG) or 25 (HG) mM glucose at 100 % confluence for 10 days with the selenium compounds. Then RNA was isolated, messengers were detected, and the data were expressed relativized with the expression of each control. **A.** Glutathione peroxidase 1, GPX1. **B.** Thioredoxin Reductase 1, TXRD1. **C**. Peroxiredoxin 1, PRDX1. **D**. Aquaporin 1, AQP1. **E**. $\alpha$-smooth muscle actin, $\alpha$-SMA. **F**. Transforming growth factor beta receptor 1, TGFβ-R1. **G**. Transforming growth factor beta receptor 2, TGFβ-R2 and **H**. Thioredoxin-1, TXN1. Bars represent average ± SE from 3 independent experiments. 18S RNA was used as the internal control. The asterisk on the bars shows significant differences found with one-way ANOVA with Bonferroni post-hoc.

**Supplemental Figure S5. Image panels at three magnifications of collagen fibers stained with Picrus Sirius Red.** After decellularization of CCD1068Sk cultures treated or not with selenium compounds in NG or HG, the ECMs were fixed and stained with Picrus Sirius Red, a specific stain for collagen. Panel **A** shows images acquired at a magnification of 40X, with the black bar in the lower right representing 500 μm. In Panel **B**, images are displayed at a magnification of 100X, with the black bar in the lower right representing 250 μm. In Panel **C**, the images have a magnification of 400X, with the black bar in the lower right representing 50 μm.

**Supplemental Figure S6. Data distribution of branches per fiber obtained from SEM of ECMs.** The distribution of the number of branches from fibers secreted by fibroblasts maintained in **A**. NG and **B**. HG in the absence and presence of 1 μM selenium compounds: Sodium selenite (SS, $Na_2SeO_3$), Selenocysteine (SeCys) and Selenomethionine (SeMet).

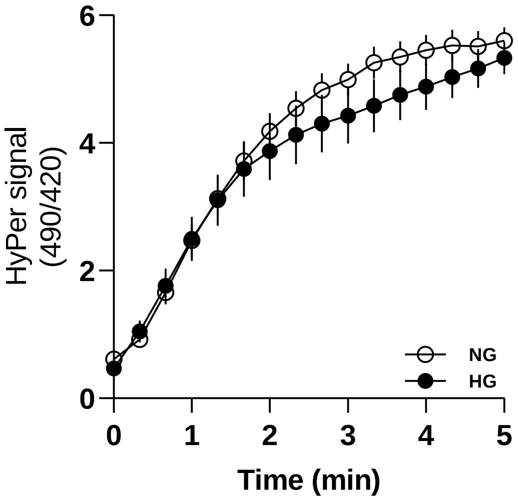


## Supplemental Figure S1

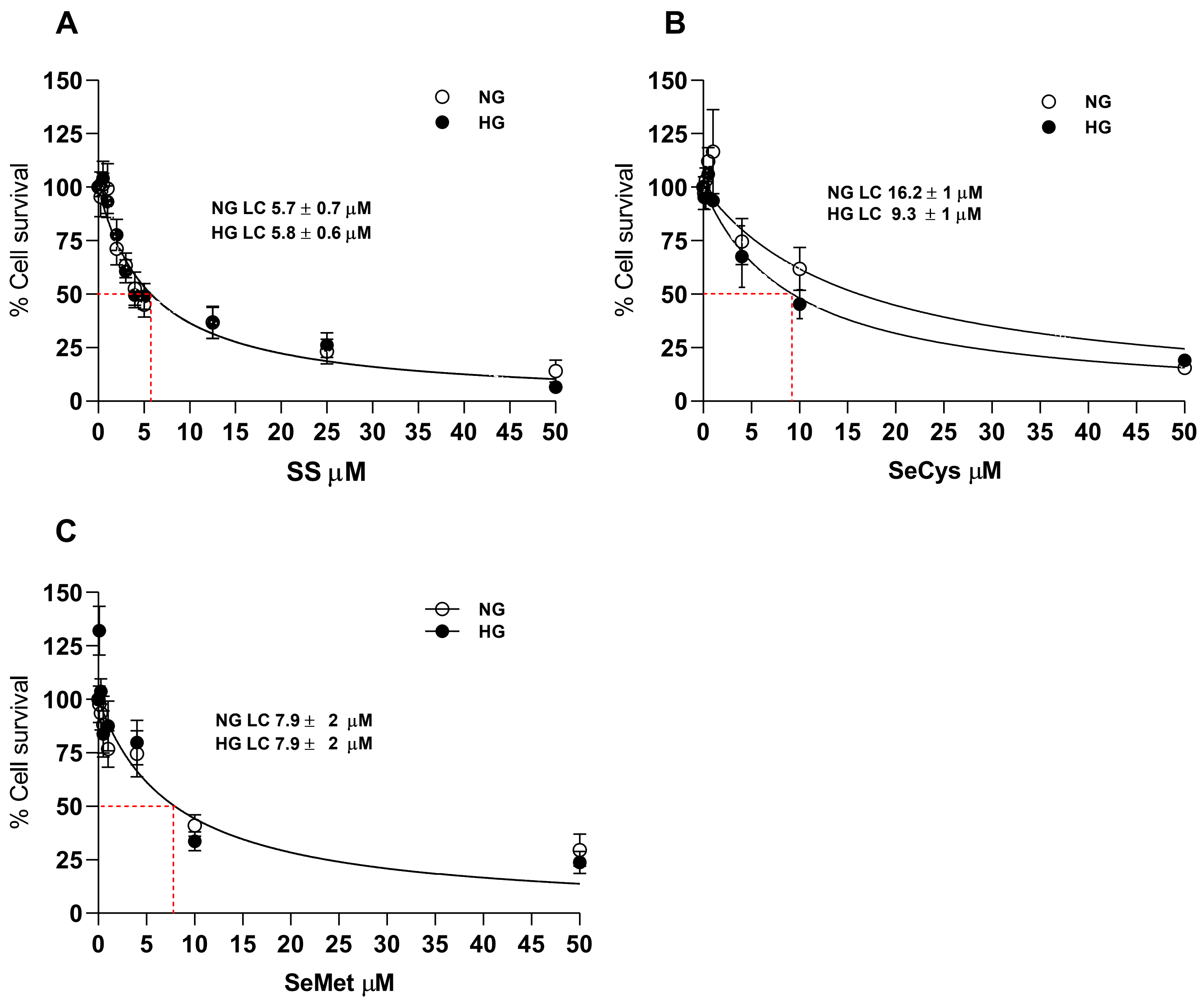


**Supplemental Figure S2**

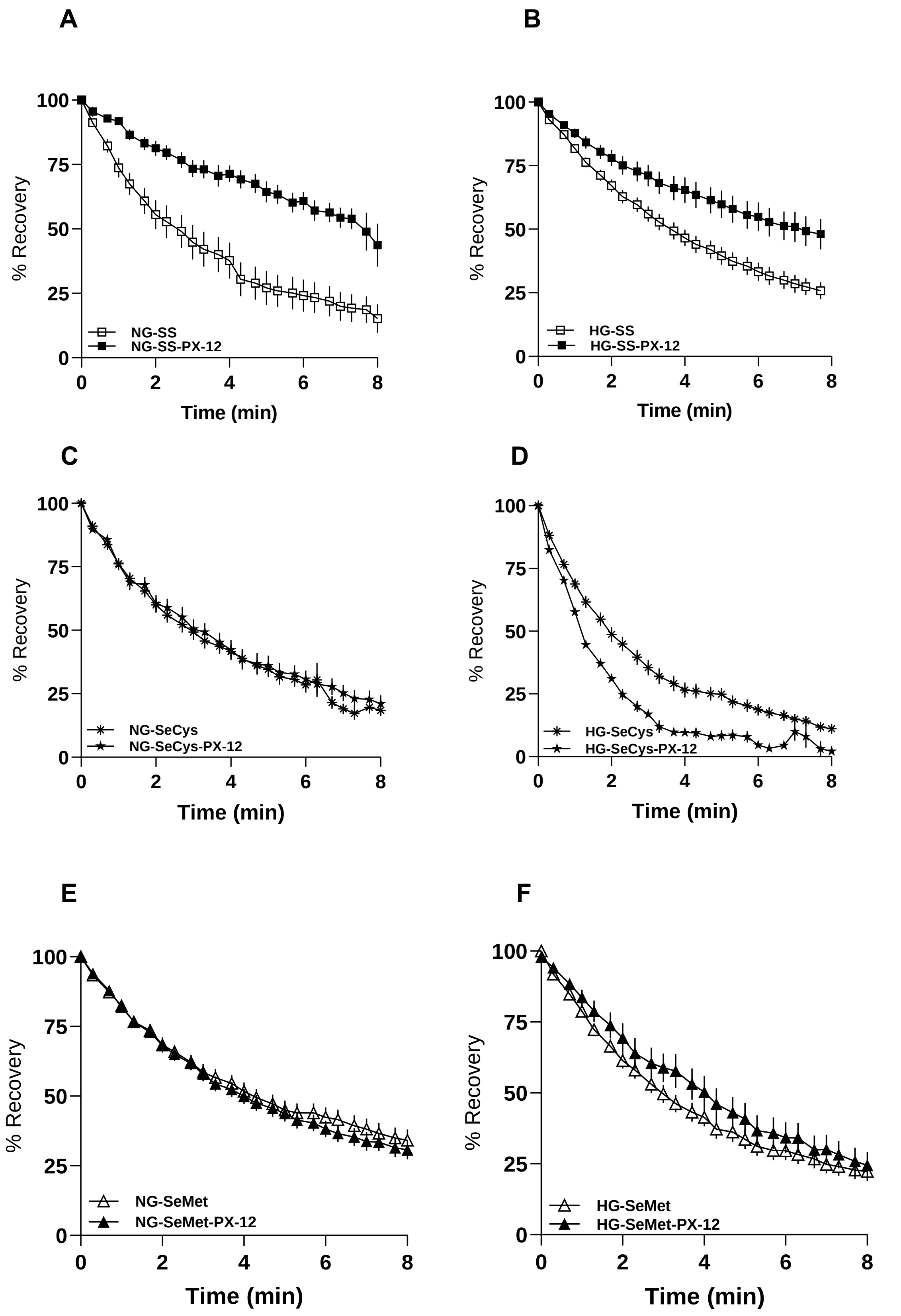


## Supplemental Figure S3

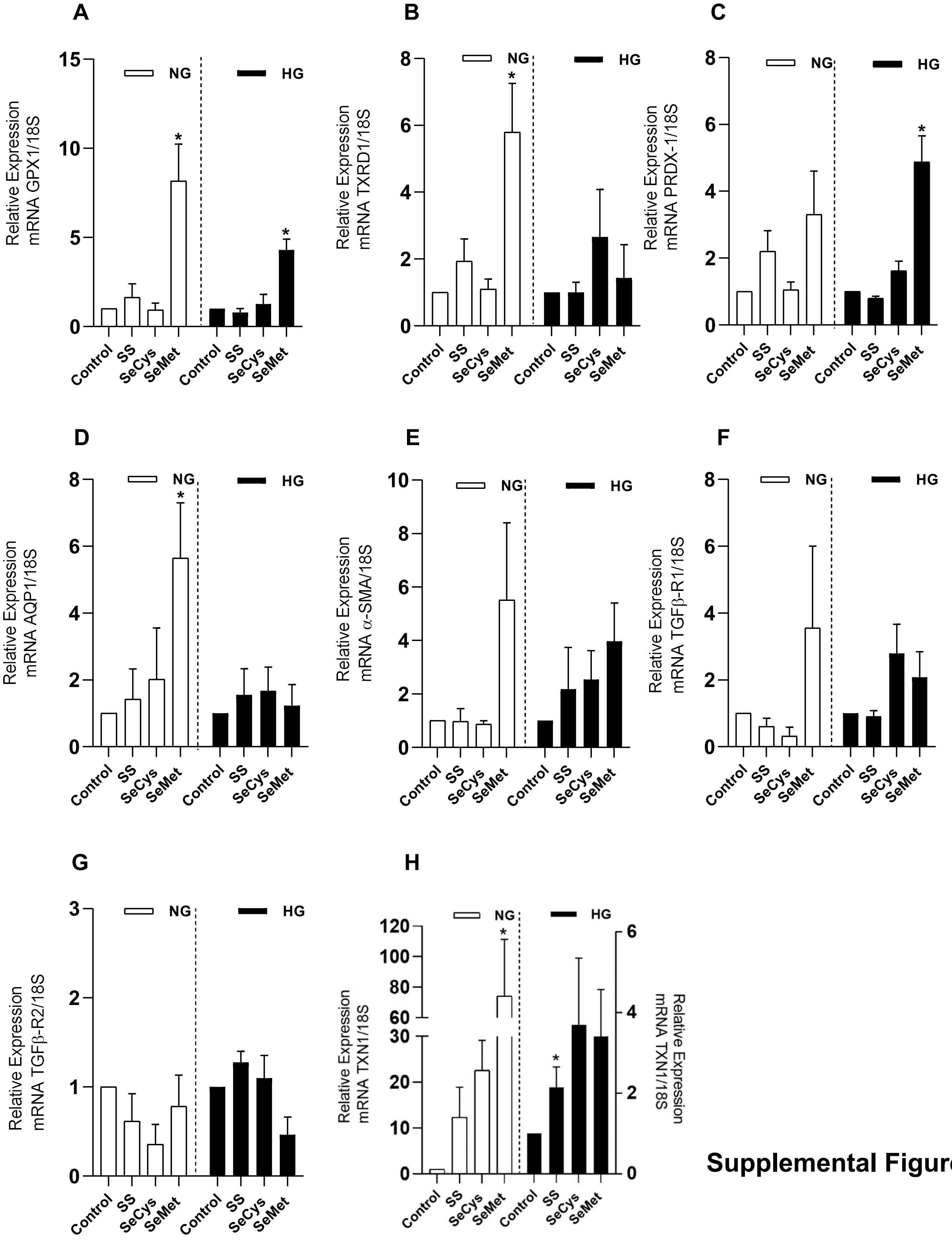


Supplemental Figure S4

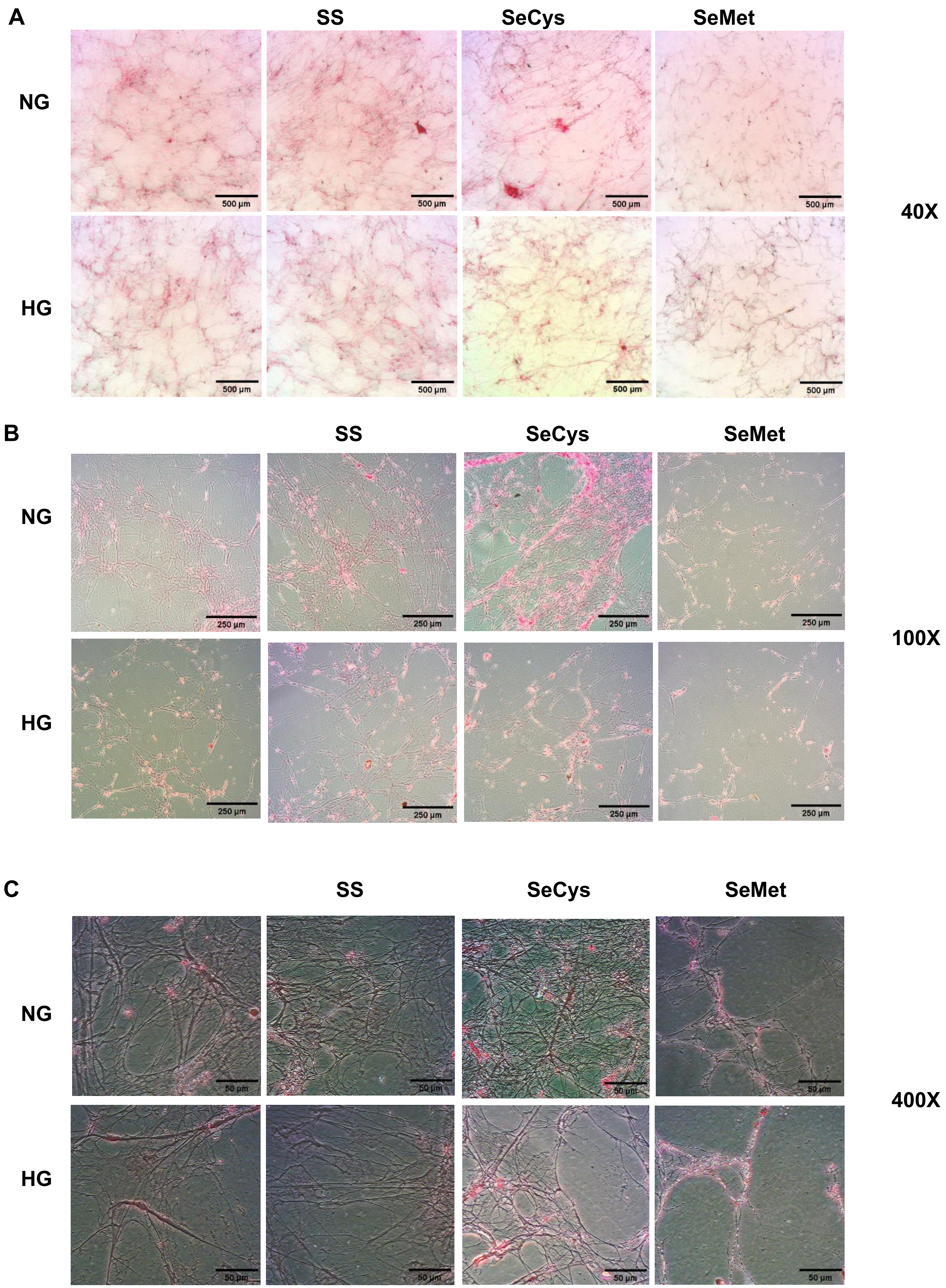


Supplemental Figure S5

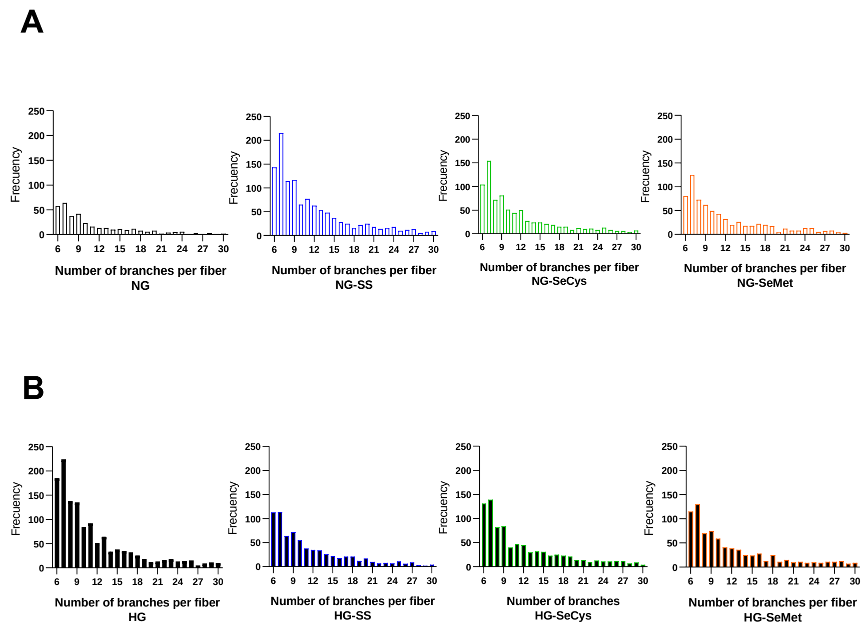


# Supplemental Figure S6
